# A Church-Based Diet and Physical Activity Intervention for Rural, Lower Mississippi Delta African American Adults: Delta Body and Soul Effectiveness Study, 2010–2011

**DOI:** 10.5888/pcd10.120286

**Published:** 2013-06-06

**Authors:** Lisa Tussing-Humphreys, Jessica L. Thomson, Tanyatta Mayo, Emanuel Edmond

**Affiliations:** Author Affiliations: Jessica L. Thomson, Tanyatta Mayo, US Department of Agriculture, Agricultural Research Service, Baton Rouge, Louisiana; Emanuel Edmond, Delta Health Alliance, Stoneville, Mississippi.

## Abstract

**Introduction:**

Obesity, diabetes, and hypertension have reached epidemic levels in the largely rural Lower Mississippi Delta (LMD) region. We assessed the effectiveness of a 6-month, church-based diet and physical activity intervention, conducted during 2010 through 2011, for improving diet quality (measured by the Healthy Eating Index-2005) and increasing physical activity of African American adults in the LMD region.

**Methods:**

We used a quasi-experimental design in which 8 self-selected eligible churches were assigned to intervention or control. Assessments included dietary, physical activity, anthropometric, and clinical measures. Statistical tests for group comparisons included χ^2^, Fisher’s exact, and McNemar’s tests for categorical variables, and mixed-model regression analysis for continuous variables and modeling intervention effects.

**Results:**

Retention rates were 85% (176 of 208) for control and 84% (163 of 195) for intervention churches. Diet quality components, including total fruit, total vegetables, and total quality improved significantly in both control (mean [standard deviation], 0.3 [1.8], 0.2 [1.1], and 3.4 [9.6], respectively) and intervention (0.6 [1.7], 0.3 [1.2], and 3.2 [9.7], respectively) groups, while significant increases in aerobic (22%) and strength/flexibility (24%) physical activity indicators were apparent in the intervention group only. Regression analysis indicated that intervention participation level and vehicle ownership were significant positive predictors of change for several diet quality components.

**Conclusion:**

This church-based diet and physical activity intervention may be effective in improving diet quality and increasing physical activity of LMD African American adults. Components key to the success of such programs are participant engagement in educational sessions and vehicle access.

## Introduction

Obesity, diabetes, and hypertension have reached epidemic levels in the largely rural Lower Mississippi Delta (LMD) region of Mississippi; African American adult residents are disproportionately affected (1). In 2011, LMD African American adults had higher prevalences for overweight and obesity (76% vs 70%), diabetes (15% vs 13%), and hypertension (48% vs 40%) than their white counterparts (2). Poor diet quality and low levels of physical activity are likely factors contributing to high disease prevalence in this region. Diet quality among LMD residents is significantly lower than in the general US population (3). Research suggests that eating patterns of LMD adults differ from patterns of adults in the rest of the United States with regard to primary food sources of nutrients and consumption of dishes using regional foods (4). Additionally, cultural norms dictate large portion sizes (5) and consumption of fried, energy-dense, and nutrient-poor foods (4). The percentage of LMD African American adults reporting no exercise in the past 30 days was higher than that of LMD white adults (47% vs 40%) (2). Comprehensive diet and physical activity interventions are needed in the LMD if these documented health disparities are to be reduced.

Churches play an important role in rural communities, serving as primary organizational units and sources of social support and leadership (6). As such, they are potentially effective settings for implementing health interventions (7,8). In this study we chose to adapt the National Cancer Institute- and American Cancer Society–sponsored Body and Soul program. This church-based dietary intervention has been extensively evaluated and shown to effectively increase fruit and vegetable consumption in African American adults (9). The primary objective of this study was to assess the effectiveness of an adaptation of this lifestyle intervention for improving diet quality and increasing physical activity among LMD African American adults. We also examined postintervention changes in anthropometric and clinical outcomes and associations among demographic characteristics, level of participation, and study outcomes.

## Methods

The procedures followed in this study were in accordance with ethical standards for human research and were approved by the institutional review board of Delta State University, Cleveland, Mississippi.

### Church recruitment, eligibility, and committee formation and responsibilities

Ten African American churches across 4 LMD counties were identified and contacted by mailed study invitation letters in May 2010. Research coordinators followed up with church pastors by telephone to gauge interest and schedule an informational study presentation following a Sunday service (during May and early June 2010). Churches were eligible if agreeable to assignment to intervention or control conditions and able to register at least 20 eligible adult members. Individual participant eligibility criteria included age (≥18 years) and not being currently pregnant. Eight churches were eligible, provided a signed letter of intent, and enrolled in the study. Churches were notified of their treatment assignment in July 2010. The other 2 churches declined to participate after the informational presentation.

Church program committees of 3 to 5 members were formed and led by the pastor and his wife, and typically included a health professional, such as a nurse or social worker; committee membership was at the discretion of the church. Intervention church committees were responsible for recruiting participants, scheduling and coordinating data collection and educational events, advertising the intervention activities, assisting with educational session presentations, and purchasing and preparing healthful foods served at the intervention functions. Control church committees recruited participants and scheduled data collection events.

### Intervention

Delta Body and Soul (DBS) is a 6-month intervention adapted from the original Body and Soul program described in detail elsewhere (9). The current intervention used many of the components of the original intervention with several modifications, including exclusion of peer counseling, broadening of dietary focus with emphasis on the regional cuisine, additional educational sessions, a didactic physical activity session, and a self-directed physical activity component (Table 1). The intervention consisted of a kickoff celebration followed by monthly, 60-minute educational sessions emphasizing increased consumption of fruits, vegetables, whole grains, and low-fat dairy foods, and decreasing solid fats, added sugars, and sodium. Information on appropriate portion sizes and the health impact of consuming the targeted foods were included in each of the dietary sessions. Healthful foods and beverages consistent with lesson themes were served at these events. One educational event was centered on benefit of, recommendations for, and strategies for overcoming barriers to physical activity. Self-directed physical activity was promoted through an adaptation of the Walking with Jesus pedometer program (10). Pedometer use was demonstrated by staff and tracking sheets were provided to record daily step counts. The educational presentation and activities (eg, label reading, cooking demonstration) were developed by the staff and were presented collaboratively with a trained church committee member. Intervention participants received a binder containing the 6 DBS lessons, healthy recipes, and other nutrition, chronic disease prevention, and physical activity–related handouts (eg, US Department of Agriculture MyPyramid materials). Intervention participants received monthly newsletters that featured nutrition and physical activity topics, healthy recipes, and testimonials from fellow church members about making diet and physical activity changes. Participants in the control churches received bimonthly newsletters containing information pertaining to colds and influenza, food safety, and minimizing stress.

**Table 1:** Comparison of Delta Body and Soul 2010–2011 Program Elements to the Body and Soul Parent Study Elements

Program Element	Delta Body and Soul Study	Body and Soul Parent Study (9)
Program length	6 months	6 months
Church committees	Formed after church commitment letter and treatment allocation was complete	Formed before intervention kickoff
	Duties included participant recruitment, event planning, and implementation of church-wide activities	Duties included planning and implementing church-wide activities
Research staff assistance	Modest, developed educational sessions and provided technical assistance	Minimal, technical assistance only
Kickoff event	Church-wide celebration	Church-wide celebration
	Pastor sermon focused on spirituality and health	Mini health fair
	Healthful eating covenant signed by pastor and displayed visibly in the church	Fruits and vegetables served
	Healthful foods prepared by church committee served	
Health screening	Baseline and postintervention	Baseline and postintervention
Educational sessions	6 sessions (monthly, 60 minutes)	At least 3 sessions
Educational session content	Fruits; vegetables; whole grains; low-fat dairy; fat, sugar, and sodium; and physical activity	Fruits and vegetables
Self-directed physical activity	Faith-based pedometer program	Not used
Peer counseling and motivational interviewing	Not used	2 telephone calls by trained lay church volunteers
Cookbook	Not used	*Eat for Life* cookbook
Other printed materials	Educational binder with 6 educational session presentations and supplemental nutrition, chronic disease prevention, and physical activity handouts (eg, US Department of Agriculture MyPlate handouts)	American Cancer Society brochures
	Monthly mailed newsletters with nutrition and physical activity information, healthy recipes, and participant testimonials	
Forgotten Miracles video	Not used (out of print)	One copy to each participant

### Data collection and measures

Baseline data were collected during July through October 2010 and postintervention during February through May 2011. Written informed consent was obtained from all participants. Questionnaire data included demographic characteristics, self-reported medical diagnoses, medications, and smoking. Physical activity was measured using the Rapid Assessment of Physical Activity (RAPA) survey (11). The RAPA allows for classification of aerobic physical activity into 1 of 5 categories — sedentary, underactive, underactive regular light, underactive regular, and active; anything less than active is considered suboptimal. The RAPA also allows for classification of strength and flexibility physical activity into 1 of 4 categories — none, strength only, flexibility only, and both strength and flexibility. Dietary intake for the previous 6 months was measured using the Delta Food Frequency Questionnaire (Delta FFQ) (4). Data obtained from the Delta FFQ were used to generate Healthy Eating Index-2005 (HEI-2005) total and component scores. HEI-2005 measures adherence to the 2005 Dietary Guidelines for Americans (2005 DGA) (12). HEI-2005 comprises 12 components summed to create a total score with a maximum value of 100 (13). For each component, higher scores reflect better adherence to 2005 DGA recommendations. Only valid (≤5 missing questionnaire items) and plausible (daily intake between 500 and 6,000 kcal) Delta FFQs were used in the analyses. Anthropometric measures included height, measured using a vertical stadiometer (Shorr Production, Olney, Maryland) and weight, measured using a calibrated digital scale (model BWB-500, Tanita Corp, Tokyo, Japan). Body mass index (BMI) was calculated as weight (kg) divided by height (meters) squared. Blood pressure was measured using an automatic blood pressure monitor (HEM780, Omron Healthcare Inc, Kyoto, Japan). Nonfasting blood lipids (high density lipoprotein, low density lipoprotein, total cholesterol, and triglycerides) and glucose were measured by using a Cholestech LDX Analyzer (Alere Inc, Waltham, Massachusetts). Intervention and control participants were provided results of their clinical outcomes at both time points. Participants who had abnormal values were advised to meet with their health care provider for further evaluation.

### Statistical analyses

Statistical analyses were performed using SAS software, version 9.2 (SAS Institute Inc, Cary, North Carolina). To test for significant group differences in the categorical measures, χ^2^ tests of association, Fisher’s exact, or McNemar’s tests were performed. General linear mixed-model regression analysis was used to test intervention effects on continuous outcomes. Church was modeled as a random effect by using a variance components covariance structure. Least squares means were computed to estimate and compare outcome changes. Tukey-Kramer adjusted *P* values were used for multiple comparisons. Group comparisons included intervention versus control and study completers versus noncompleters. Study completers were participants who provided both baseline and postintervention measures; noncompleters were participants who did not provide postintervention measures. Because exploratory analyses indicated that participation level had an effect on some outcome changes, the intervention group was subdivided into 2 categories — low participation (attendance at <4 sessions) and high participation (attendance at ≥4 sessions). Selection of outcomes included in multivariable analysis was based on differences between intervention and control groups, or between low- and high-participation intervention subgroups (*P* < .10). Covariates used in the analysis included treatment (control, low-, and high-participation intervention); age; sex; employment status; vehicle ownership; current smoking status; baseline outcome measurement (ie, diet quality and physical activity variables), BMI, and diagnoses of hypertension and diabetes. Employment status was categorized as unemployed, employed (full and part-time), or retired; aerobic physical activity as suboptimal (sedentary, underactive, underactive regular light, and underactive regular) or optimal (active); and strength/flexibility physical activity as some (strength, flexibility, and both) or none. The significance level of the tests was set at .05.

## Results

For both control (n = 208) and intervention (n = 195) groups, most participants were female, obese, nonsmokers; owned a vehicle; and were either employed or retired (Table 2). Mean age was 46 years in the control group and 47 years in the intervention group. At baseline, the intervention participants were significantly more likely to have a self-reported diagnosis of high cholesterol (23% vs 11%), take hypoglycemic agents (79% vs 53%), have lower diastolic blood pressure (DBP) (12% vs 13%), and have a higher HEI-2005 total score (11% vs 9%) compared with control participants.

**Table 2:** Baseline Characteristics for and Comparisons Between Control (N = 208) and Intervention Participants (N = 195), Delta Body and Soul, Mississippi, 2010–2011^a^

Characteristic	Control, n (%)	Intervention, n (%)	*P *Value
**Sex**			
Female	144 (69.2)	148 (76.3)	.11^b^
Male	64 (30.8)	46 (23.7)	
**Weight group** c			
Healthy weight	28 (13.5)	28 (14.9)	.78^b^
Overweight	51 (24.6)	41 (21.8)	
Obese	128 (61.8)	119 (63.3)	
**Current smoker**			
No	132 (81.5)	134 (86.5)	.23^b^
Yes	30 (18.5)	21 (13.5)	
**Own vehicle**			
No	46 (23.8)	39 (21.0)	.50^b^
Yes	147 (76.2)	147 (79.0)	
**Employment status**			
Employed	95 (46.1)	107 (54.9)	.06^b^
Retired	33 (16.0)	36 (18.5)	
Unemployed	78 (37.9)	52 (26.7)	
**Chronic disease**			
Diabetes	35 (16.9)	38 (19.5)	.50^b^
Hypertension	106 (51.2)	110 (56.4)	.30^b^
Cardiovascular disease	16 (7.7)	15 (7.7)	.99^b^
High cholesterol	23 (11.1)	44 (22.6)	.002^b^
**Medication use**			
Have diabetes but do not take medication	15 (46.9)	7 (20.6)	.02^b^
Have diabetes and take medication for it	17 (53.1)	27 (79.4)	
Have hypertension but do not take medication	40 (44.0)	44 (44.0)	.99^b^
Have hypertension and take medication for it	51 (56.0)	56 (56.0)	
**Aerobic physical activity^d^**			
Sedentary	21 (10.3)	12 (6.5)	.85^b^
Underactive	12 (5.9)	8 (4.3)	
Underactive regular light	30 (14.7)	33 (17.7)	
Underactive regular	30 (14.7)	30 (16.1)	
Active	111 (54.4)	103 (55.4)	
**Strength/flexibility physical activity^d^**			
Neither	112 (55.4)	95 (51.6)	.45^b^
Strength	19 (9.4)	13 (7.1)	
Flexibility	34 (16.8)	31 (16.8)	
Both	37 (18.3)	45 (24.5)	
**Anthropometric and clinical**			
Age, y, mean (SD)	46 (16.8)	47 (15.3)	.57^e^
BMI, kg/m^2^, mean (SD)	34 (8.6)	34 (9.0)	.82^e^
Systolic blood pressure, mm Hg, mean (SD)	138 (22.7)	134 (22.1)	.21^e^
Diastolic blood pressure, mm Hg, mean (SD)	83 (12.9)	79 (11.9)	.03^e^
High density lipoprotein cholesterol, mg/dL, mean (SD)	52 (15.9)	50 (16.6)	.31^e^
Low density lipoprotein cholesterol, mg/dL, mean (SD)	113 (42.6)	105 (33.6)	.21^e^
Total cholesterol, mg/dL, mean (SD)	189 (46.7)	187 (41.7)	.75^e^
Triglycerides, mg/dL, mean (SD)	135 (96.8)	160 (104.8)	.06^e^
Glucose, mg/dL, mean (SD)	113 (52.9)	113 (51.0)	.95^e^
Healthy Eating Index-2005 total score, mean (SD)	52 (8.6)	57 (11.2)	.04^e^

Abbreviations: BMI, body mass index; SD, standard deviation.

a Not all categories add up to 208 or 195 participants because of missing data. Data are presented as n (%) unless otherwise stated.

b *P* value for between-group difference based on χ^2^ or Fisher’s exact tests.

c Healthy weight was defined as BMI of 18.5–24.0 kg/m^2^ (included 1 underweight participant); overweight, 25.0–29.0 kg/m^2^; obese, ≥30 kg/m^2^.

d Measured using the Rapid Assessment of Physical Activity (RAPA) survey (11). For aerobic physical activity, anything less than active is considered suboptimal.

e *P* value for between-group difference based on mixed-model regression analysis.

Retention rates were 85% (176 of 208) for the control group and 84% (163 of 195) for the intervention group. Study completers were significantly more likely to own a vehicle (81% vs 59%; *P* < .001), have self-reported diagnoses of hypertension (57% vs 36%; *P* < .001) and arthritis (22% vs 9%; *P* < .01), and were significantly older (48 vs 39 years; *P* < .001) at baseline compared with noncompleters. Of participants with dietary data at both baseline and postintervention, 76% (241 of 318) had valid and plausible Delta FFQs. Mean educational class attendance was 2.6 (standard deviation [SD], 2.2) sessions; 59% of the intervention participants attended at least 3 of the 6 educational sessions.

Significant increases in the mean HEI-2005 total fruit, total vegetable, dark green and orange vegetables and legumes (DGOV&L), and total scores occurred in both the intervention and control groups (range, 0.2 to 3.4 points; Table 3); there were no significant differences between groups (ie, no intervention effects). No changes were significant in anthropometric and clinical outcomes for either group. Significant changes in both self-reported aerobic (*P* = .02) and strength/flexibility (*P* = .03) physical activity outcomes were apparent in the intervention group only. Of intervention participants reporting changes in activity levels from baseline to postintervention, significantly more reported increased (optimal) aerobic and increased strength/flexibility physical activity (22% and 24%) compared with decreased physical activity (11% and 13%). Pedometer logs were available for only a few participants; therefore, these data were not analyzed.

**Table 3:** Mean Changes and Tests for Within- and Between-Group Changes^a^ in Outcomes, Delta Body and Soul, Mississippi, 2010–2011

Outcome	Control		Intervention		*P *Value^b^	*P *Value^c^
	n	Mean (SD)	n	Mean (SD)		
**Healthy Eating Index-2005**						
Total fruit	120	0.3 (1.8)	121	0.6 (1.7)	.01	.33
Whole fruit	120	0.6 (1.8)	121	0.3 (1.8)	.08	.24
Total vegetables	120	0.2 (1.1)	121	0.3 (1.2)	.03	.35
DGOV&L	120	0.3 (0.9)	121	0.3 (1.3)	.04	.83
Total grains	120	0.1 (0.9)	121	0 (0.8)	.82	.61
Whole grains	120	0.3 (1.5)	121	0.3 (1.6)	.16	.86
Milk	120	0.2 (2.6)	121	0.1 (2.4)	.74	.74
Meat and beans	120	0 (1.5)	121	−0.1 (1.5)	.68	.67
Oils	120	0.1 (2.6)	121	0.3 (3.0)	.41	.65
Saturated fat	120	0.5 (3.2)	121	0.5 (3.3)	.27	.83
Sodium	120	0.2 (2.7)	121	−0.2 (2.5)	.44	.33
SoFAAS	120	0.6 (4.7)	121	0.7 (4.8)	.22	.92
Total	120	3.4 (9.6)	121	3.2 (9.7)	.01	.86
**Anthropometric and clinical**						
Weight, kg	176	0.1 (5.8)	156	−0.7 (9.4)	.31	.39
Body mass index, kg/m^2^	175	0.0 (2.1)	156	−0.2 (3.3)	.30	.36
Systolic blood pressure, mm Hg	174	0.9 (18.0)	163	−0.7 (17.3)	.74	.98
Diastolic blood pressure, mm Hg	174	−2.2 (9.9)	163	−1.1 (9.4)	.31	.59
High density lipoprotein cholesterol, mg/dL	169	−2.2 (9.6)	158	0.5 (10.5)	.50	.20
Low density lipoprotein cholesterol, mg/dL	143	−9.6 (28.8)	137	−3.0 (23.0)	.45	.14
Total cholesterol, mg/dL	174	−9.8 (29.4)	161	−2.9 (25.4)	.83	.15
Triglycerides, mg/dL	172	6.6 (93.0)	160	−4.4 (96.5)	.58	.33
Glucose, mg/dL	174	−6.2 (44.3)	160	−5.4 (49.1)	.21	.87

Abbreviations: SD, standard deviation; DGOV&L, dark green and orange vegetables and legumes; SoFAAS, solid fats, alcoholic beverages, and added sugars.

a Change = 6 months follow-up findings − baseline findings.

b *P* value for within-group change based on mixed-model regression analysis.

c *P* value for between-group difference in change based on mixed-model regression analysis.

The 2 significant continuous predictor variables were age and corresponding baseline outcome value. Age was significant only for HEI-2005 total score; every 1 year decrease in age was associated with a 0.1-point increase in score. Corresponding baseline values were significant predictors of change for all selected outcomes; effects ranged from 0.5 to 0.8 unit increases for every 1 unit decrease in baseline values.

For treatment effects, increases in all reported outcomes were significant in the high participation intervention group; significant increases in the control group were apparent for HEI-2005 whole fruit and total scores only (Table 4). Increases ranged from 0.4 points (HEI-2005 total vegetables and DGOV&L scores) to 7.4 points (HEI-2005 total score) in the high-participation intervention group. Female sex was a significant positive predictor of change for HEI-2005 whole grains and oils scores. Vehicle ownership was a significant positive predictor of change for HEI-2005 total vegetables and DGOV&L scores. Postintervention change in HEI-2005 solid fats, alcoholic beverages, and sugars (SoFAAS) score was significantly greater for participants who owned a vehicle compared with participants without a vehicle. However, within-group changes failed to reach significance. Similarly, between group change in the HEI-2005 oils score was significantly greater for employed and retired participants compared with unemployed participants. Again, the within-group changes were not significant. No other outcome models were significant nor were any other covariates significant predictors of postintervention change for the models.

**Table 4:** Mixed-Model Regression Results for Selected Outcome Changes,^a^ Delta Body and Soul, Mississippi, 2010–2011

Outcome^c^	Least Squares Means (SE) for Categorical Predictor Variables^b^									
	Treatment			Sex		Own Vehicle		Employment Status		
	Control	Low Participation Intervention	High Participation Intervention	Female	Male	No	Yes	Unemployed	Employed	Retired
Whole fruit^d^	**0.4** (0.12)	0.1 (0.18)	**0.9** (0.18)	—e	—e	—e	—e	—e	—e	—e
Total vegetables	−0.1 (0.10)	0 (0.13)	**0.4** (0.14)	—e	—e	−0.1 (0.15)	**0.3** (0.07)	—e	—e	—e
DGOV&L	0 (0.13)	0.1 (0.15)	**0.4** (0.17)	—e	—e	0 (0.16)	**0.4** (0.09)	—e	—e	—e
Whole grains	0 (0.14)	−0.1 (0.18)	**0.6** (0.19)	**0.4** (0.11)	−0.1 (0.17)	—e	—e	—e	—e	—e
Oils^f^	−0.2 (0.28)	−0.1 (0.33)	**0.9** (0.35)	**0.5** (0.22)	−0.1 (0.31)	—e	—e	−0.4 (0.30)	0.4 (0.24)	0.6 (0.36)
SoFAAS	−0.6 (0.50)	−0.8 (0.58)	**1.5** (0.63)	—e	—e	−0.8 (0.62)	0.8 (0.36)	—e	—e	—e
Total^g^	**1.5** (0.73)	1.4 (1.07)	**7.4** (1.12)	—e	—e	—e	—e	—e	—e	—e

Abbreviations: SE, standard error; DGOV&L, dark green and orange vegetables and legumes; SoFAAS, solid fats, alcoholic beverages, and added sugars.

a Change = 6 months follow-up findings − baseline findings.

b Values in bold type indicate change is significantly different from zero and from nonbold values (*P* ≤ .05 for familywise error rate); exceptions noted in footnotes d and f.

c Measured by the Healthy Eating Index-2005 (13).

d For treatment comparisons, difference between low and high participation intervention group means is significant (*P* ≤ .05 for familywise error rate); difference between control and high participation intervention group means is trending for significance (.05 < *P* ≤ .10 for familywise error rate).

e The independent (predictor) variable was not included in the model because it was not significant. Least squares means cannot be calculated for variables not included in models.

f For treatment comparisons, difference between control and high participation intervention group means is significant (*P* ≤ .05 for familywise error rate); difference between control and low participation intervention group means is not significant (*P* > .05 for familywise error rate).

g Employed and retired group means were significantly greater than unemployed group mean (*P* ≤ .05 for familywise error rate).

## Discussion

The primary objective of this study was to assess the effectiveness of a 6-month, church-based, lifestyle intervention to improve diet quality and increase physical activity in African American adults residing in the LMD region of Mississippi. Significant improvements were apparent for some components of diet quality. However, these improvements were observed in both the intervention and control group, indicating a lack of intervention effect. Significant increases in both aerobic physical activity and strength/flexibility physical activity were apparent in the intervention, but not the control group. Similar results were observed in another church-based study conducted in urban, southern, African American adults (14).

A plausible explanation for lack of a significant dietary intervention effect could be the level of participation. Total and 6 components of diet quality improved significantly in the high-participation intervention subgroup but not in the control group (except for whole fruit and total scores) or low participation intervention subgroup. These results are encouraging because 5 of the components — whole fruit, total vegetables, DGOV&L, whole grains, and SoFAAS — were topics of the educational sessions. Similar results were observed in diet quality measures in the DBS pilot study. In an evaluation of the original Body and Soul program, researchers found that event attendance was significantly associated with increased fruit and vegetable consumption (15). Cumulatively, results highlight the positive effect of attendance at educational sessions. Unfortunately, group interventions do not allow for a high degree of scheduling flexibility, which can negatively affect overall attendance and intervention dose (16,17).

Although our study was not specifically designed to achieve improvements in anthropometric and clinical outcomes, we did assess changes in these outcomes. Overall, none of the changes in the anthropometric and clinical outcomes were significant for the intervention or control churches. However, exploratory analyses involving participation level revealed that clinically meaningful decreases of 4.4 and 2.8 mm Hg in SBP and DBP, respectively, were observed in the high-participation intervention group. Similar blood pressure decreases were reported in 2 separate community-based physical activity interventions conducted in Mississippi African American adults (18,19).

An interesting finding was the positive effect of vehicle ownership on study completion and attendance at intervention activities. Lack of personal vehicle ownership and public transportation are common in economically deprived, rural communities (20). Rural residents often travel considerable distances to attend church activities (21). Anecdotally, participants without vehicles reported difficulty in attending weekly church activities, including our educational sessions. Lack of access to vehicles and rising gas prices in spring 2011 (22) were reasons given for nonparticipation. Although DBS classes were often planned on the same day as other church activities, the intervention schedule could not accommodate all participants. Other studies have reported lack of personal transportation as a barrier to attendance at intervention activities (23,24). In this study, improvements in vegetable consumption were detected only in participants who owned a vehicle. Limited vehicle access and considerable distance to food stores have been reported as barriers to fruit and vegetable consumption in African American and rural-dwelling adults (25). Access to transportation should be considered when designing dietary interventions in rural, economically depressed regions.

This study has several strengths, including intervention in a community with elevated chronic disease prevalence, inclusion of both men and women, and modest attrition rates. This study also has several limitations. The self-report nature of the Delta FFQ and RAPA could have introduced bias in outcomes because of poor recall or socially desirable responses. Underreporting of food intake is common, especially among obese people (26). Although pedometer tracking sheets were provided to the intervention participants, data were unreliable, and an objective measure of physical activity was lacking. Conclusions regarding independent effects of participation level and vehicle ownership are difficult to draw because of confounding. An additive effect for level of attendance and vehicle ownership is likely because both variables were significant predictors of diet quality. Generalizability of the results is limited because the intervention was tailored specifically for African American adults in the LMD region and they were predominately older, churchgoing, and female. Overrepresentation of older women in LMD African American churches is common and reflects the low percentage of active male church members. Finally, selection bias may have occurred because churches providing a letter of intent may have been motivated to make health behavior changes and hence not represent all African American churches in the LMD region of Mississippi.

The results of DBS suggest that a church-based diet and physical activity intervention can improve diet quality and increase physical activity in the target population. Key components to successful programs are attendance at the educational sessions and access to transportation. Future interventions must address the complexity of individual-level behaviors with socioenvironmental barriers that make healthy eating and physical activity challenging for rural residents.
